# Supplementary material for: Mechanical self-adaptive porous valve relying on surface tension for energy harvesting from low-flux bubbles
Source: Nat Commun. 2025 Nov 22;16:11544. doi: 10.1038/s41467-025-66500-6 (PMC12749621; doi:10.1038/s41467-025-66500-6)
Supplement: Supplementary file 1 — Supplementary Information [file 41467_2025_66500_MOESM1_ESM.pdf]

Supplementary Information

Mechanical self-adaptive porous valve relying on surface tension for energy harvesting from low-flux bubbles

Yu Du (杜宇)[1, *], Ping Li (李平)[2, 3], Yumei Wen (文玉梅)[2, 3], Yunsheng Fan (范云生)[1], and Zhichen Liu (刘志晨)[1]

1. College of Marine Electrical Engineering, Dalian Maritime University, Dalian, Liaoning, 116026, China.
2. School of Automation and Intelligent Sensing, Shanghai Jiao Tong University, Shanghai, 200240, China.
3. State Key Laboratory of Submarine Geoscience, Shanghai Jiao Tong University, Shanghai, 200240, China

*Corresponding author. E-mail: yudu@dlmu.edu.cn

Table of contents:

1. Structural composition of the porous valve....2
2. Bubble contact angle of the porous plate....2
3. Principle of bubble energy harvesting....2
4. Experimental setup for bubble energy harvesting....4
5. Measurement of the output gas flux of the bubble releaser....5
6. Analysis of the energy conversion process....6
7. Discussion about the cone half-angle of the micropores....8
    7.1 Bubble-accumulation stage....8
    7.2 Bubble-release stage....10
    7.3 Comparative experiment with different cone half-angles....10
8. Porous valves with different pore diameters....11
    8.1 Performance of bubble release....11
    8.2 Output current and electrical energy....12
9. Output current and electrical energy at different gas fluxes....13
10. Calculation of energy harvesting efficiency....13
11. Estimate of electricity generation potential....14
12. Stability performance....14
    12.1 External fluid disturbances....14
    12.2 Bubble plume impingement....16

1. Structural composition of the porous valve

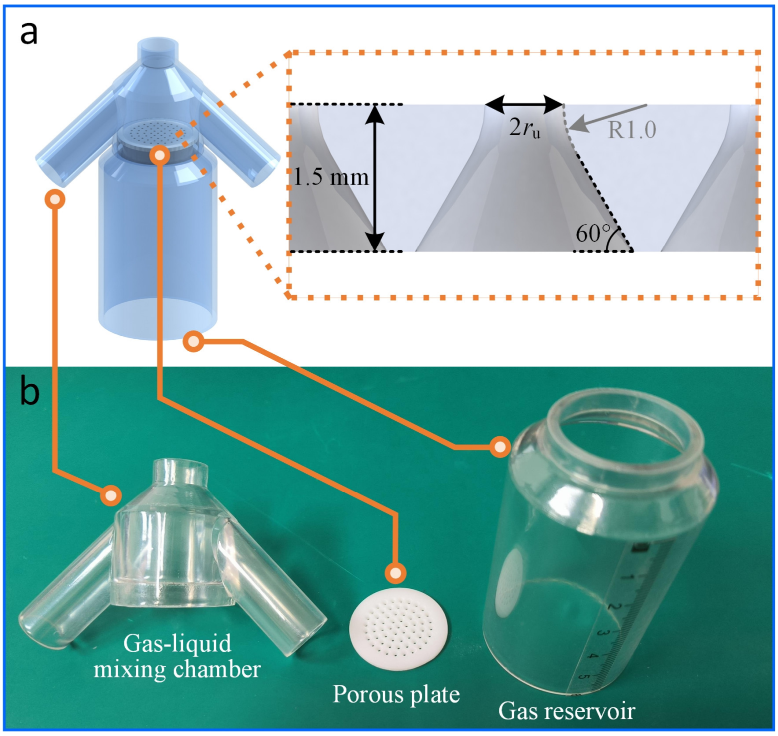

Figure S1 Structural parameters and components of the mechanical self-adaptive porous valve. (a) Diagram of structural parameters. r_u represents the radius of the upper rim of the micropore. (b) Photograph of the components.

2. Bubble contact angle of the porous plate

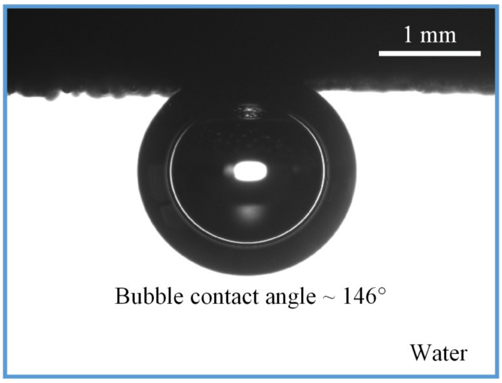

Figure S2 Bubble contact angle of the porous plate in seawater.

3. Principle of bubble energy harvesting

The working principle of the bubble energy harvesting device based on the mechanical self-adaptive porous valve is shown in Figure S3. The gas-liquid mixing chamber of the porous valve, the bubble rising pipe, and the external water environment form a complete internal and external

circulation loop of water flow. The water inlets on both sides of the gas-liquid mixing chamber of the porous valve are the openings through which water from the external environment enters the energy harvesting device. The water outlet above the turbine generator is the opening through which water returns from the device to the external water environment. These inlets and outlets are essential, as they ensure the smooth circulation of the loop and the rapid upwelling of the gas-liquid two-phase fluid in the bubble rising pipe.

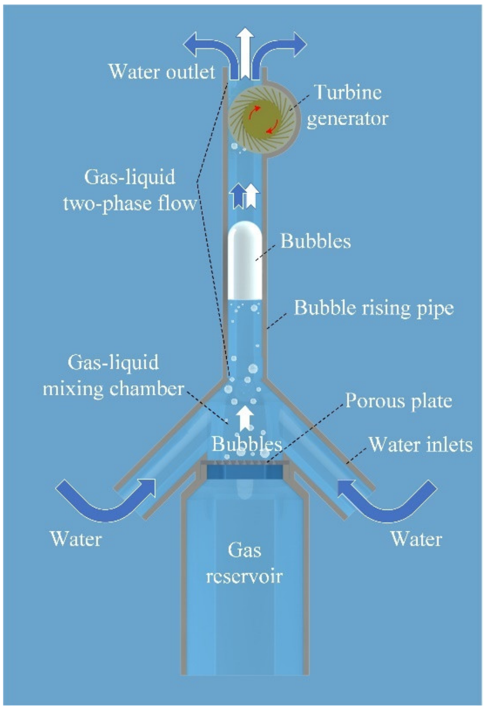

Figure S3 Working principle of the bubble energy harvesting device.

The working process of the bubble energy harvesting device is as follows. After the bubbles accumulated in the gas reservoir are released through the porous plate, they start to float upward and enter the bubble rising pipe. The bubbles rise in the pipe to form an upwelling flow, leading to a higher flow rate in the pipe than that in the external water environment (which can be approximated as a quasi-static liquid). According to Bernoulli's principle, the fluid pressure inside the device is much lower than that in the external water environment, thereby generating a pressure difference at the water inlets on both sides of the porous valve. Due to the pressure difference, water from the external water environment is drawn into the pipe through the water inlets. At the same time, the rising gas-liquid two-phase fluid in the pipe flows through the turbine and exits from the outlet at the upper end of the pipe, returning to the external water environment, thus forming a complete water circulation loop. When the fast-rising gas-liquid two-phase flow in the pipe passes through the turbine, it generates a strong driving force to drive the turbine generator to rotate and output electrical energy. In this circulation of water flow, the buoyancy of the bubbles does positive work on the water in the pipe. The buoyancy potential energy of the bubbles is converted into the kinetic energy of the gas-liquid two-phase fluid, which is finally converted into electrical energy by the turbine generator and output to the external load. It should be noted that due to the liquid surface tension, the porous plate inside the porous valve can only

allow bubbles to pass through but not water flow. If the water inlets and outlets are not set, the water circulation inside and outside the bubble rising pipe cannot be realized. Accordingly, the bullet-shaped bubbles occupying the entire pipe diameter can only rise slowly within the pipe or even get stuck, leading to blockage. Such blockage can hinder the formation of upwelling flow, thereby incapacitating the system from supplying adequate fluid power for the rotation of the turbine generator.

4. Experimental setup for bubble energy harvesting

The experimental setup for underwater low-flux bubble energy harvesting is shown in Figure S4. The bubble energy harvesting device (BEHD) consists of a transparent bubble rising pipe and a turbine generator. The bubble rising pipe with the turbine generator installed at its upper end is immersed in water. The length and inner diameter of the pipe are 0.8 m and 8 mm, respectively. The mechanical adaptive porous valve is installed at the lower end of the pipe. A bubble releaser placed on the bottom of the water is connected to a variable-speed gas pump through a silicone hose. The gas pump is driven by a stepper motor (57HS5630D8EI, Sumtor Wuxi Electric Equipment Co., Ltd.). The rotation rate of the motor is controlled by the frequency of the square wave signal output from a function signal generator (DG1022Z, RIGOL Technologies Co., LTD.), which is used to adjust the gas flux. The bubble releaser releases bubbles into the water through a stainless-steel needle. The outer diameter of the needle is 0.63 mm, and the inner diameter is 0.39 mm. The needle outputs bubbles with a diameter of about 3 mm, which is similar to the size of the submarine methane bubbles, simulating a subsea bubble seepage.

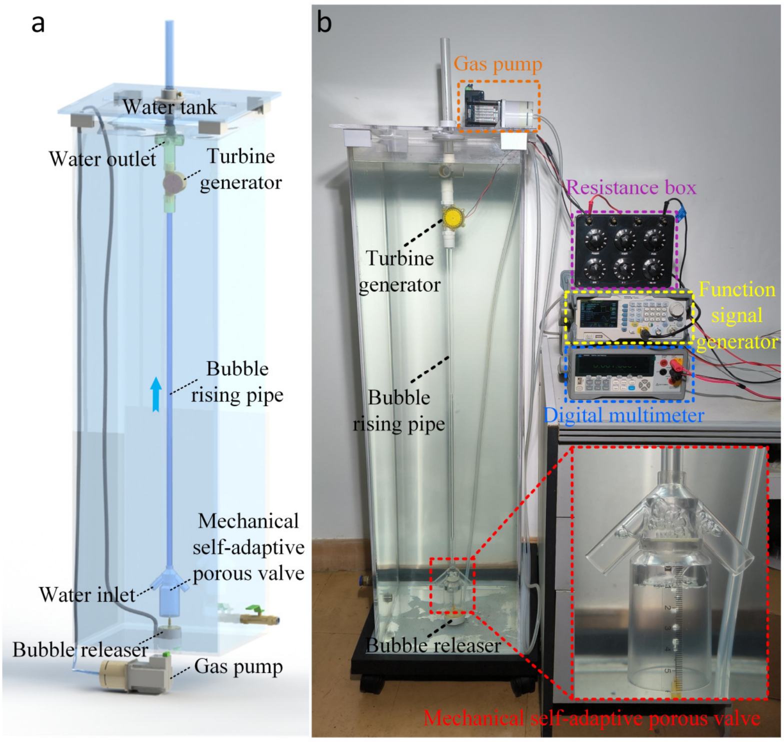

Figure S4 Experimental setup for low-flux bubble energy harvesting based on the mechanical self-adaptive porous valve. (a) Schematic diagram. (b) Photograph.

5. Measurement of the output gas flux of the bubble releaser

To ensure the repeatability of the experimental conditions, the output gas flux of the bubble releaser for different rotation rates of the gas pump is measured. The experimental setup is in the inset of Figure S5(a). An inverted 100-mL measuring cylinder is suspended in water for measuring the volume of the gas released by the bubble releaser. At the beginning of the experiment, the measuring cylinder is filled with water. As the released bubbles enter the measuring cylinder, the liquid level in the measuring cylinder gradually falls. The volume of gas released by the bubble releaser as a function of time for different rotation rates of the gas pump is plotted in Figure S5(a). At any constant rotation rate, the gas volume increases linearly with time, which proves that the output gas flux of the bubble releaser is constant. The bubble releaser as well as the gas pump has favorable stability, which can meet the needs of repeated experiments. According to the results in Figure S5(a), the average gas flux of the bubbles released by the bubble releaser for different rotation rates of the gas pump is calculated and listed in Figure S5(b). The gas flux on the abscissa of Figures 5(a) and 5(g) in the main text is derived from the results in Figure S5(b).

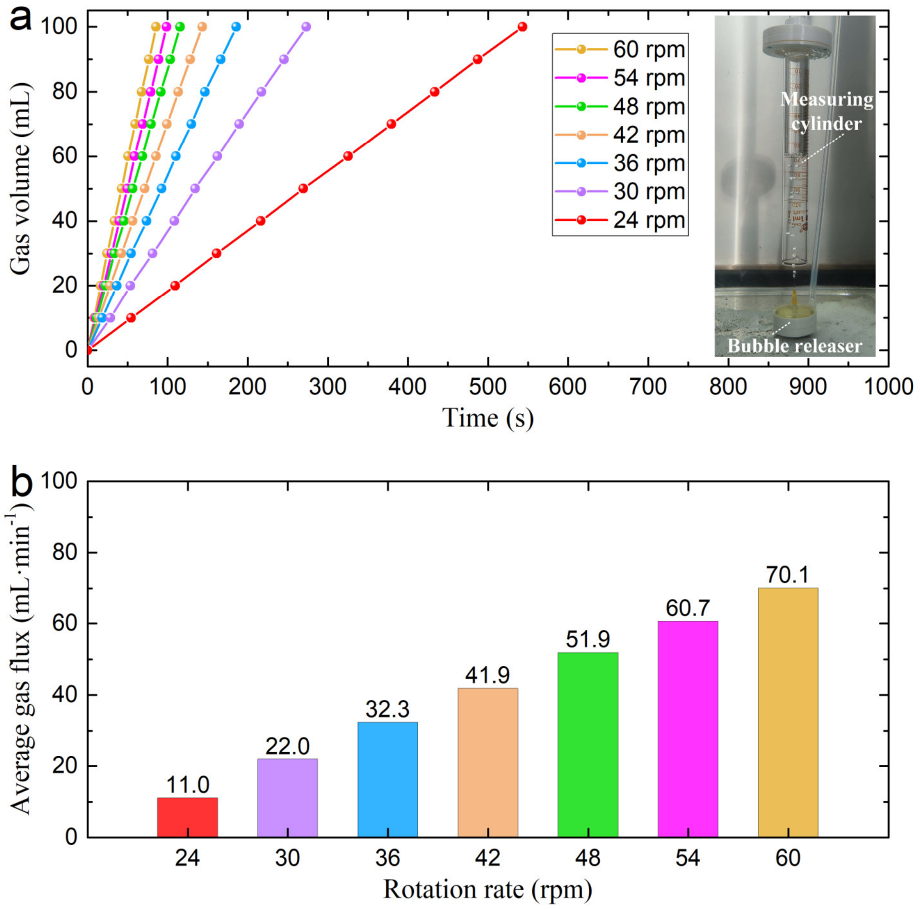

Figure S5 Output gas flux of the bubble releaser for different rotation rates of the gas pump. (a) Cumulative output gas volume of the bubble releaser for different rotation rates of the gas pump. The inset is a photograph of the experimental setup. (b) Average gas flux of the bubbles released by the bubble releaser for different rotation rates of the gas pump.

6. Analysis of the energy conversion process

In the bubble energy harvesting system, as shown in Figure S3, the buoyancy of bubbles drives the fluid circulation inside and outside the device, and the fluid power drives the turbine generator to rotate and output electrical energy. The main function of the system is to convert the buoyancy potential energy of bubbles into electrical energy. The specific energy conversion process is as follows. When bubbles rise in the pipe, the buoyancy of the bubbles does work on the gas-liquid two-phase fluid in the pipe, driving the fluid to flow upward. The buoyancy potential energy E_b of the bubbles is released, with part converted into the kinetic energy E_k of the gas-liquid two-phase fluid, and the rest consumed by overcoming various resistive work, including the viscous resistance loss E_v, the frictional resistance loss E_r from turbine rotation, and the electrical energy E_e generated by overcoming the electromagnetic resistance of the generator. Accordingly, the energy conversion equation of the bubble energy harvesting system is

$$-\Delta E_b = \Delta E_k + E_v + E_r + E_e, \tag{S1}$$

where ΔE_b and ΔE_k represent the variations of the buoyancy potential energy E_b and the kinetic energy E_k, respectively. ΔE_b takes a negative value because the release of buoyancy potential energy occurs as bubbles ascend.

When the rising distance of bubbles is short and the change in hydrostatic pressure acting on the bubbles is insignificant, the variation in bubble volume caused by the pressure change is negligible. During a period t_1, the amount of buoyancy potential energy released by the bubble is equal to the work done by the buoyancy force, which can be expressed as

$$-\Delta E_b = F_b L = \rho g Q_g t_1 L = \rho g V_g L, \tag{S2}$$

where F_b represents the buoyancy force of bubbles, L represents the length of the vertical pipe, ρ is the liquid density, g is the acceleration of gravity, Q_g is the gas-intake flux of the pipe, and V_g is the total volume of bubbles entering the bubble energy harvesting device over a period t_1. Driven by the gas-liquid two-phase flow, the turbine generator produces an induced electromotive force of an induced electromotive force of

$$e = NBSk\omega_t \sin k\omega_t t, \tag{S3}$$

where N represents the number of turns in the generator coil, B denotes the magnetic flux density, S is the effective area of the coil, k is the number of magnetic pole pairs, ω_t is the angular velocity of turbine rotation, and t represents time. The resulting loop current is

$$i = \frac{e}{R_{in} + R_{ex}} = \frac{NBSk\omega_t \sin k\omega_t t}{R_{in} + R_{ex}}, \tag{S4}$$

where R_{in} denotes the internal resistance of the energy harvester, and R_{ex} is the external load resistance. Correspondingly, the output power of the turbine generator is

$$P_{out} = i^2 R_{ex} = \frac{R_{ex}\left(NBSk\omega_t\right)^2 \sin^2 k\omega_t t}{\left(R_{in} + R_{ex}\right)^2} \tag{S5}$$

The average output power over the corresponding sine period is

$$P_{ave} = \frac{R_{ex}\left(NBSk\omega_t\right)^2}{2\left(R_{in} + R_{ex}\right)^2} \tag{S6}$$

When the gas-liquid two-phase fluid in the bubble rising pipe is in a stable flow state, and both the flow rate of the fluid and the rotation rate of the turbine generator remain constant, the electrical energy output by the bubble energy harvesting device over a period t_1 is

$$E_e = P_{ave} t_1 = \frac{R_{ex}\left(NBSk\omega_t\right)^2 V_g}{2\left(R_{in}+R_{ex}\right)^2 Q_g} \tag{S7}$$

The output energy density of the bubble energy harvesting device (electrical energy obtained from unit volume of bubbles) is

$$E_d = \frac{E_e}{V_g} = \frac{n_t^2}{Q_g} \cdot \frac{2\pi^2 R_{ex}\left(NBSk\right)^2}{\left(R_{in}+R_{ex}\right)^2} = \lambda \cdot \frac{2\pi^2 R_{ex}\left(NBSk\right)^2}{\left(R_{in}+R_{ex}\right)^2}, \tag{S8}$$

where $n_t = \omega_t / (2\pi)$ denotes the rotational speed (number of revolutions per second) of the turbine. Equation (S8) indicates that the output energy density of the bubble energy harvesting device is proportional to the square of the turbine rotational speed n_t and inversely proportional to the gas-intake flux Q_g of the bubble rising pipe. $\lambda = n_t^2/Q_g$ can be defined as the rotational speed-flux coefficient. According to Equation (S8), the following conclusion can be drawn: Increasing the rotational speed-flux coefficient of the bubble energy harvesting system contributes to enhancing the output energy density of the bubble energy harvesting device.

To validate the correctness of the above theoretical analysis, quantitative experimental studies were conducted on the rotational speed-flux coefficient and output energy density of the bubble energy harvesting device at different gas-intake fluxes. In this experiment, to facilitate the adjustment and control of the gas-intake flux of the pipe, the bubble energy harvesting device was not equipped with the porous valve, and the bubbles directly entered the bubble rising pipe without accumulation. The data on gas-intake flux are derived from the experimental results in Figure S5(b). The number of revolutions of the turbine is calculated based on the number of cycles of the sinusoidal voltage signal output by the generator to the load resistance (35 Ω). The experimental results are listed in Table S1.

Table S1 Rotational speed-flux coefficient and output energy density at different gas-intake fluxes

Gas-intake flux Q_g (mL·min^{-1})	Number of revolutions of the turbine N (r)	Average rotational speed of the turbine n_t (r·s^{-1})	Electrical energy output E (mJ)	Rotational speed-flux coefficient λ (r^2·m^{-3}·s^{-1})	Energy density E_d (mJ·L^{-1})
51.9	112	0.37	0.12	1.61×10^5	0.46
60.7	252	0.84	0.46	6.97×10^5	1.52
70.1	347	1.16	0.85	1.15×10^6	2.43
78.3	416	1.39	1.22	1.47×10^6	3.12
87.1	459	1.53	1.48	1.61×10^6	3.40

As shown in Table S1, both the turbine rotational speed n_t and the rotational speed-flux coefficient λ of the bubble energy harvesting device increase with the increase in the gas-intake flux Q_g of the pipe. For example, when the gas-intake flux of the pipe is 87.1 mL·min^{-1}, the average rotational speed and rotational speed-flux of the energy harvesting device are 1.53 r·s^{-1} and 1.61×10^6 r^2·m^{-3}·s^{-1}, respectively, some 4.14 and 10 times that (0.37 r·s^{-1} and 1.61×10^5 r^2·m^{-3}·s^{-1}) at the gas-intake flux of 51.9 mL·min^{-1}. Since the rotational speed-flux coefficient λ is proportional to the square of the turbine rotational speed n_t and inversely proportional to the gas-

intake flux Q_g, the increase in turbine rotational speed n_t has a more significant effect on enhancing λ than the increase in Q_g. By virtue of the increase in the rotational speed-flux coefficient λ, the output energy density of the device increases from 0.46 mJ·L^{-1} at 51.9 mL·min^{-1} to 3.40 mJ·L^{-1} at 87.1 mL·min^{-1}.

These theoretical analyses and experimental results indicate that when the gas-intake flux of the bubble rising pipe increases, the electrical energy harvested from unit volume of bubbles also increases. In other words, when the total volume V_g of bubbles entering the bubble energy harvesting device is constant, increasing the gas-intake rate of the pipe contributes to enhancing the power generation and average output power of the device. Based on this principle, the mechanical self-adaptive porous valve proposed in this paper is used to accumulate low-flux bubbles and release them into the bubble rising pipe at high speed, thereby significantly increasing the instantaneous gas-intake rate of the pipe. This not only leads to a significant enhancement in the instantaneous output power of the bubble energy harvesting device, but also contributes to the improvement of its average output power.

7. Discussion about the cone half-angle of the micropores

7.1 Bubble-accumulation stage

During the bubble-accumulation stage, the interfacial forces acting on the gas in the mechanical self-adaptive porous valve maintain dynamic equilibrium. The Laplace pressure difference $\Delta p = 2\gamma / R$ across the curved gas-liquid interface within the micropores is equal in magnitude to the liquid pressure difference $p_d - p_u = \rho g \Delta h$ between the upper and lower gas-liquid interfaces of the gas, from which it follows that

$$R = \frac{2\gamma}{\rho g \Delta h}, \tag{S9}$$

where R is the radius of curvature of the gas-liquid interface, γ represents the surface tension of the liquid, ρ denotes the liquid density, g is the acceleration of gravity, and Δh is the liquid-level difference between the upper and lower gas-liquid interfaces of the gas. As shown in Figure S6, the angle between the generatrix of the micropore and the vertical axis (cone half-angle) is α. The angle between the curved gas-liquid interface and the solid-liquid interface is θ. The three-phase contact line between the gas-liquid interface and the inner wall of the micropore is a circle with radius r. According to geometric relations, it can be derived that

$$R = \frac{r}{\cos(\alpha + \theta)} \tag{S10}$$

The functional relationship between the radius r of the three-phase contact line and the liquid-level difference Δh can be derived by substituting Equation (S10) into Equation (S9), as

$$r = \frac{2\gamma \cos(\alpha + \theta)}{\rho g \Delta h} \tag{S11}$$

The variation in the radius r of the three-phase contact line reflects the positional variation of the gas-liquid interface within the micropore, where a decrease in r indicates that the interface

moves upward. According to Equation (S11), as the liquid level difference Δh gradually increases, the radius r of the three-phase contact line adaptively decreases. The corresponding result is that the gas-liquid interface and the three-phase contact line in the micropore adjust their positions adaptively and move upward. During the upward movement of the gas-liquid interface, r and $\alpha+\theta$ decrease. According to Equation (S10), the curvature radius R of the gas-liquid interface consequently decreases and gradually approaches the radius r of the three-phase contact line.

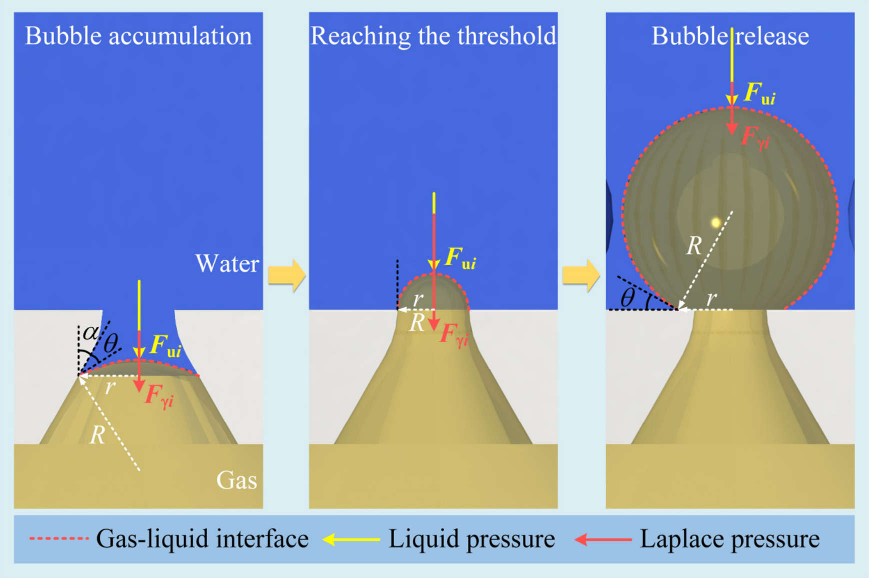

Figure S6 Mechanical analysis of the gas-liquid interface at the micropores during the process of bubble accumulation and release. The red dashed line represents the gas-liquid interface. The yellow arrows represent the liquid pressure F_{ui}. The red arrows represent the Laplace pressure $F_{\gamma i}$. R is the radius of curvature of the gas-liquid interface. r is the radius of the three-phase contact line. α is the angle between the generatrix of the micropore and the vertical axis (cone half-angle). θ is the angle between the curved gas-liquid interface and the solid-liquid interface.

The above theoretical analysis indicates that during the bubble-accumulation stage, the volume of gas accumulated in the porous valve determines the magnitude of the liquid level difference Δh, and Δh determines the liquid pressure difference $\rho g\Delta h$ exerted on the gas. To maintain dynamic mechanical equilibrium, the gas-liquid interface in the micropore adaptively adjusts its curvature radius R, thereby generating a Laplace pressure difference $2\gamma/R$ that balances the liquid pressure difference $\rho g\Delta h$. According to Equation (S9), the curvature radius R of the gas-liquid interface is completely determined by the liquid level difference Δh and is independent of the cone half-angle α of the micropore. Therefore, α does not affect the curvature radius R of the gas-liquid interface and the Laplace pressure difference $2\gamma/R$. Moreover, considering that the radius r of the three-phase contact line and the curvature radius R of the gas-liquid interface are related by the geometric Equation (S10), the radius r of the three-phase contact line is determined not only by the curvature radius R but also influenced by the cone half-angle α of the micropore. It means that the position of the gas-liquid interface depends not only on Δh but also on α. Therefore, changing the cone half-angle α of the micropores on the porous plate can

only alters the specific position of the curved gas-liquid interface within the micropores during the process of bubble accumulation, without affecting the curvature radius of the gas-liquid interface and the Laplace pressure difference.

7.2 Bubble-release stage

As shown in Figure S6, when the three-phase contact line coincides with the upper port of the micropore and the gas-liquid interface takes a hemispherical shape, the curvature radius R of the gas-liquid interface reaches the minimum value, which is equal to the radius r_u of the upper port of the micropore ($R_{min} = r = r_u$), and is independent of the cone half-angle α of the micropore. At this moment, the accumulated gas reaches the opening threshold Δh_T of the porous valve

$$\Delta h_T = \frac{2\gamma}{\rho g r_u} \tag{S12}$$

The porous valve starts to enter the bubble-release stage.

During the bubble release stage, as shown in Figure S6, the bubbles on the upper surface of the porous plate expand rapidly, and the three-phase contact line expands quickly toward the periphery of the micropore, causing the radius r of the three-phase contact line to increase rapidly. According to the geometric relationship, the curvature radius of the gas-liquid interface is

$$R = \frac{r}{\sin\theta} \tag{S13}$$

In this stage, the Laplace pressure difference across the gas-liquid interface is

$$\Delta p = \frac{2\gamma \sin\theta}{r}, \tag{S14}$$

which is determined by the angle θ between the gas-liquid interface and the solid-liquid interface and the radius r of the three-phase contact line, but independent of the cone half-angle α of the micropore.

Based on the above theoretical analyses, both in the bubble-accumulation stage and the bubble-release stage, the curvature radius R of the gas-liquid interface, the Laplace pressure difference Δp, and the opening threshold Δh_T of the porous valve are all independent of the cone half-angle α of the micropore. The influence of the cone half-angle α is only reflected in changing the specific position of the curved gas-liquid interface in the micropore during the bubble-accumulation stage, without affecting the opening threshold of the porous valve and the processes of bubble accumulation and release.

7.3 Comparative experiment with different cone half-angles

To further verify the results of the theoretical analysis, four porous plates with different cone half-angles were fabricated and installed inside the porous valve to experimentally test the threshold characteristics of the porous valve. In the experiment, all four porous plates have the same micropore upper port diameter d_u = 1.5 mm, with identical quantity and arrangement of micropores. The only difference among the four porous plates is the cone half-angle α of the micropores.

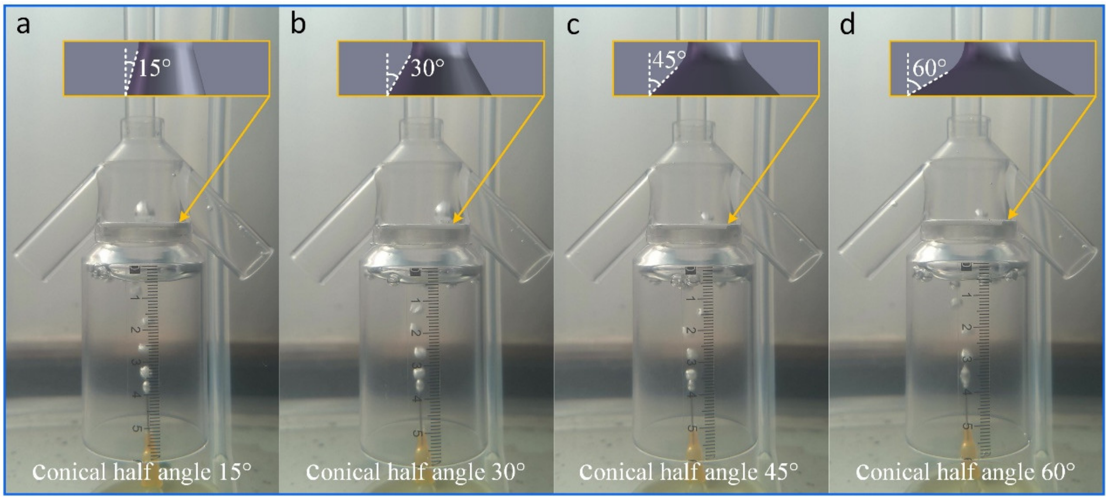

Figure S7 Experimental results of the comparison of opening thresholds for the porous valves with different cone half-angles. (a) 15°. (b) 30°. (c) 45°. (d) 60°.

As shown in Figure S7, the cone half-angles of the micropores on the four porous valves are 15°, 30°, 45°, and 60°, respectively. When the gas accumulated in the four porous valves reaches the thresholds, the scales corresponding to the gas-liquid interface below the gas are all between 4 mm and 5 mm. This indicates that the opening thresholds of all porous valves fall within the range of 15.5–16.5 mm, which is close to the experimental result of 16.0 mm for the porous valve with a 1.5-mm pore diameter in Figures 4(a) and (b) of the main text. The experimental results further confirm that the threshold characteristics of the porous valve are independent of the cone half-angle of the micropores.

8. Porous valves with different pore diameters

8.1 Performance of bubble release

In the main text, Figure 4e shows the variation trend of the gas-release rate of the porous valves with pore diameter at an environmental gas flux of 70.1 mL·min^{-1}. To minimize the influence of the gas-intake behavior on the process of bubble release, eight repetitive experiments were conducted on the porous valves with different pore diameters under the minimum gas flux (11.0 mL·min^{-1}) provided by the experimental setup in Figure S4. The corresponding gas-release rates are presented in Table S2. The experimental results show that the average gas-release rate of porous valves during the bubble-release stage still exhibits an increasing trend with the increase in pore diameter.

Table S2 Gas-release rate during the bubble-release stage of the porous valves with different pore diameters in each cycle at a low environmental gas flux of 11.0 mL·min^{-1} (data unit: L·min^{-1})

Pore diameters d_u (mm)	1st	2nd	3rd	4th	5th	6th	7th	8th	Mean value	Standard deviation
0.8	1.12	1.13	1.20	1.15	1.05	1.25	1.05	1.14	1.14	0.07
1.0	1.19	1.21	1.35	1.13	1.15	1.39	1.47	1.56	1.30	0.15
1.3	1.56	1.43	1.46	1.46	1.36	1.49	1.49	1.58	1.48	0.07
1.5	1.95	2.03	1.66	1.90	1.87	1.95	1.87	1.98	1.90	0.11
1.7	2.26	2.14	2.26	2.06	1.94	2.03	2.19	1.96	2.10	0.12

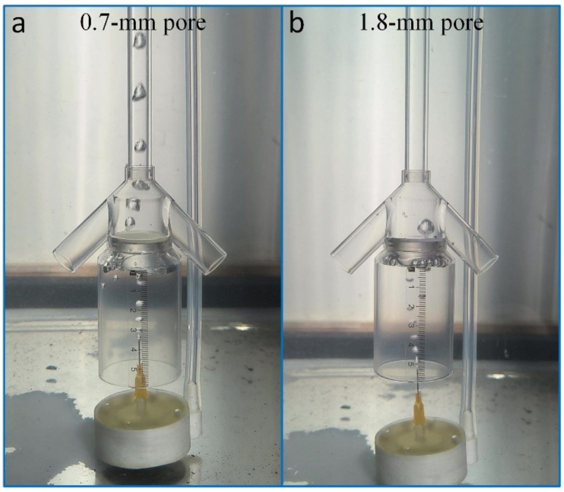

Figure S8 Photographs of bubble-release behavior of the porous valves with pore diameters of 0.7 mm and 1.8 mm. (a) The porous valve with a pore diameter of 0.7 mm releases bubbles from only a single pore. (b) Photograph of the porous valve with a pore diameter of 1.8 mm at the opening threshold, where the gas-liquid interface below the accumulated gas does not reach the scale markings range of the gas reservoir.

8.2 Output current and electrical energy

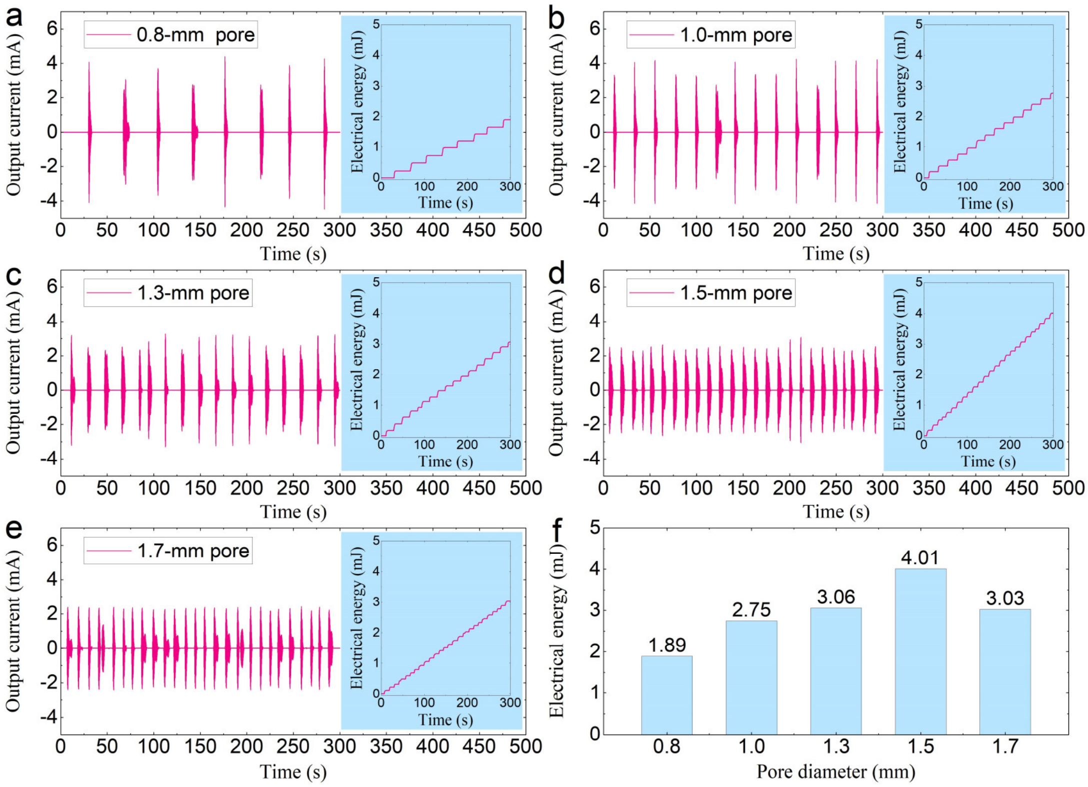

Figure S9 Output current and electrical energy of the bubble energy harvesting devices equipped with the porous valves with different pore diameters. (a) Porous valves with 0.8-mm pores. (b) Porous valves with 1.0-mm pores. (c) Porous valves with 1.3-mm pores. (d) Porous valves with 1.5-mm pores; (e) Porous valves with 1.7-mm pores. (f) Electrical energy output by the devices equipped with the porous valves with different pore diameters over 300 s.

9. Output current and electrical energy at different gas fluxes

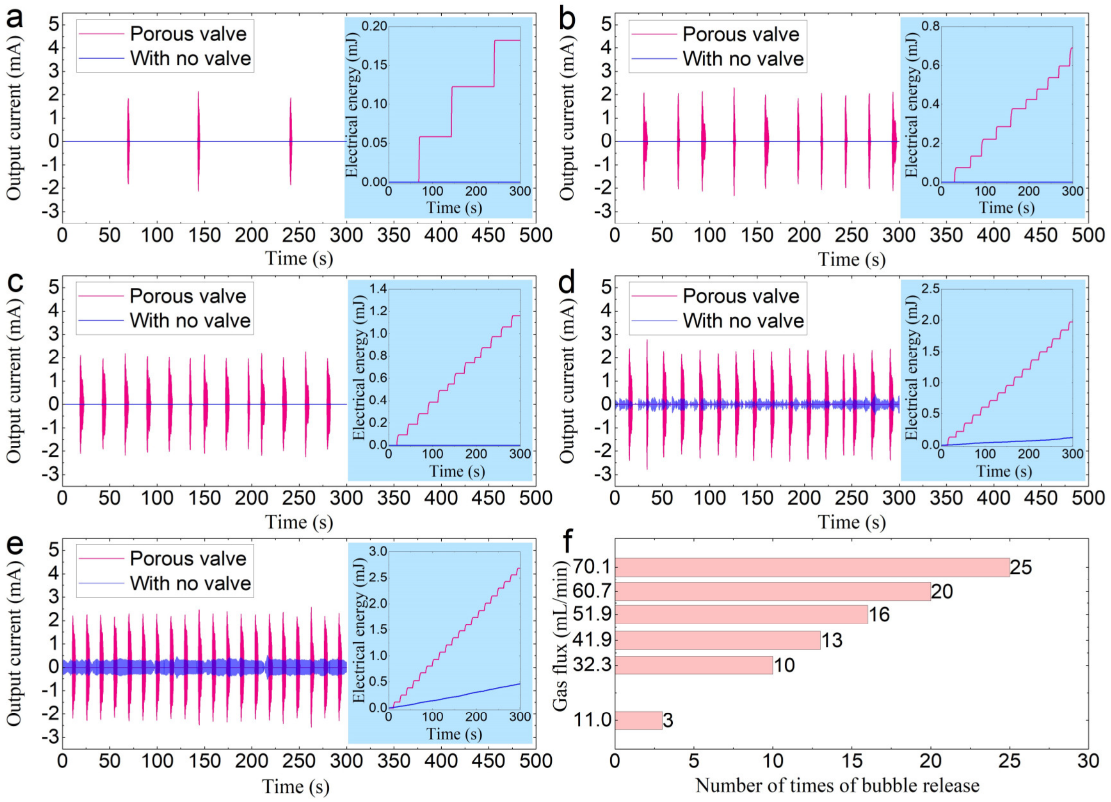

Figure S10 Output current and electrical energy of the bubble energy harvesting device equipped with a porous valve and the corresponding device with no valve when the gas flux is (a) 11.0 mL·min^{-1}, (b) 32.3 mL·min^{-1}, (c) 41.9 mL·min^{-1}, (d) 51.9 mL·min^{-1}, (e) 60.7 mL·min^{-1}. (f) Number of times of bubble release at different gas fluxes.

10. Calculation of energy harvesting efficiency

The energy conversion efficiency from bubble buoyancy potential energy E_b to electrical energy E_e is

$$\eta = \frac{E_e}{-\Delta E_b} \times 100\% = \frac{E_e}{\rho g V_g L} \times 100\%, \tag{S15}$$

where E_e represents the generated electrical energy, ΔE_b represent the variation of the buoyancy potential energy E_b, ρ is the liquid density, g is the acceleration of gravity, V_g is the total volume of bubbles entering the bubble energy harvesting device over a period t_1, and L represents the length of the vertical bubble rising pipe. In the experiment corresponding to Figure 5 in the main text, at a gas flux of 70.1 mL·min^{-1}, the total volume V_g of bubbles entering the pipe within 300 s is 350.5 mL. The electrical energy E_e output by the bubble energy harvesting device with a porous valve and the valveless device is 4.01 mJ and 8.54×10^{-1} mJ, respectively. The seawater density ρ = 1.025×10^{3} kg·m^{-3}, acceleration of gravity g = 9.8 m·s^{-2}, and the height of the bubble rising

pipe $L = 0.8$ m. According to Equation (S15), the energy harvesting efficiencies of the bubble energy harvesting device equipped with the porous valve and the valveless device are calculated to be 0.14% and 0.03%, respectively.

11. Estimate of electricity generation potential

For a bubble with volume V_0 released from a seabed at a depth h_0 below the sea surface, according to the ideal gas equation, when the temperature and the amount of substance are constant, the gas pressure of the bubble is inversely proportional to its volume. During the ascent of the bubble in water, the gas pressure equilibrates with the surrounding seawater pressure. The decrease in the surrounding seawater pressure causes the gas pressure within the bubble to gradually decrease, leading to the expansion of the bubble volume. When the bubble rises to a water depth h, its volume $V(h)$ can be expressed as

$$V(h) = \frac{(\rho g h_0 + p_{\text{atm}})V_0}{\rho g h + p_{\text{atm}}}, \tag{S16}$$

where $\rho = 1.025\times10^3$ kg·m^{-3} is the density of seawater around the bubble, $g = 9.8$ m·s^{-2} is the acceleration of gravity, and $p_{\text{atm}} = 1.01\times10^5$ Pa is the atmospheric pressure. For the bubble energy harvesting device based on the porous valve, bubbles rise in the pipe in the form of slug flow shown in Figure 5b of the main text, each being a long baculiform bubble sufficient to fill the pipe diameter. The bubbles only contact water at the front and rear ends, leading to low mass and heat transfer efficiencies. Therefore, dissolution loss and temperature change of bubbles during ascent in the pipe are negligible. According to the results in Table 1 of the main text, the output energy density (the electrical energy obtained from a unit volume of bubbles) of the bubble energy harvesting device equipped with the porous valve is 11.44 mJ·L^{-1} within a bubble rising height of 0.8 m. Accordingly, the expected electrical energy generated by a bubble with volume V_0 rising from an underwater depth h_0 to the sea surface is

$$E_{\text{e}} = \int_{h_0}^{0} \frac{E_{\text{d}}}{0.8} V(h)\,\text{d}(h_0 - h) \tag{S17}$$

Substituting Equation (S16) into Equation S17 gives

$$E_{\text{e}} = \frac{1.25 E_{\text{d}} \left(\rho g h_0 + p_{\text{atm}}\right) V_0}{\rho g} \ln\left(1 + \frac{\rho g h_0}{p_{\text{atm}}}\right) \tag{S18}$$

Taking a bubble seepage point in the cold-spring activity area of Site F in the South China sea as an example to estimate the power generation potential. The bubble seepage point is located on the 1120 m deep seafloor, with a gas flux of 6.75 mL·min^{-1}, and the annual bubble release volume can be calculated to be approximately 3.55×10^3 L. By substituting the relevant data (h_0 = 1120 m and $V_0 = 3.55\times10^3$ L) into Equation (S18), the annual power generation is calculated to be 2.71×10^5 J.

12. Stability performance

12.1 External fluid disturbances

To further demonstrate the ability of the pipe bubble energy harvesting system based on the porous valve to resist external fluid disturbances, the performance of the device under external

water flow impact was tested by experiment. As shown in Figure S11(a), an underwater motor-driven propeller was installed on the sidewall of the experimental water tank to generate turbulent disturbances in the external water environment of the bubble energy harvesting device. During the experiment, the rotation of the underwater propeller vigorously agitated the water around the bubble energy harvesting device, causing a large number of microbubbles to be entrained into the water and follow the turbulent motion. Meanwhile, as shown in Figure S11(b), the rotation of the propeller also caused the water surface to undergo vigorous undulating motion. Under a gas flux of 70.1 mL·min^{-1}, the motion of the bubble in the bubble rising pipe of the energy harvesting device and the output current of the turbine generator under external turbulent disturbances are presented in Figures S11(c) and (d), respectively.

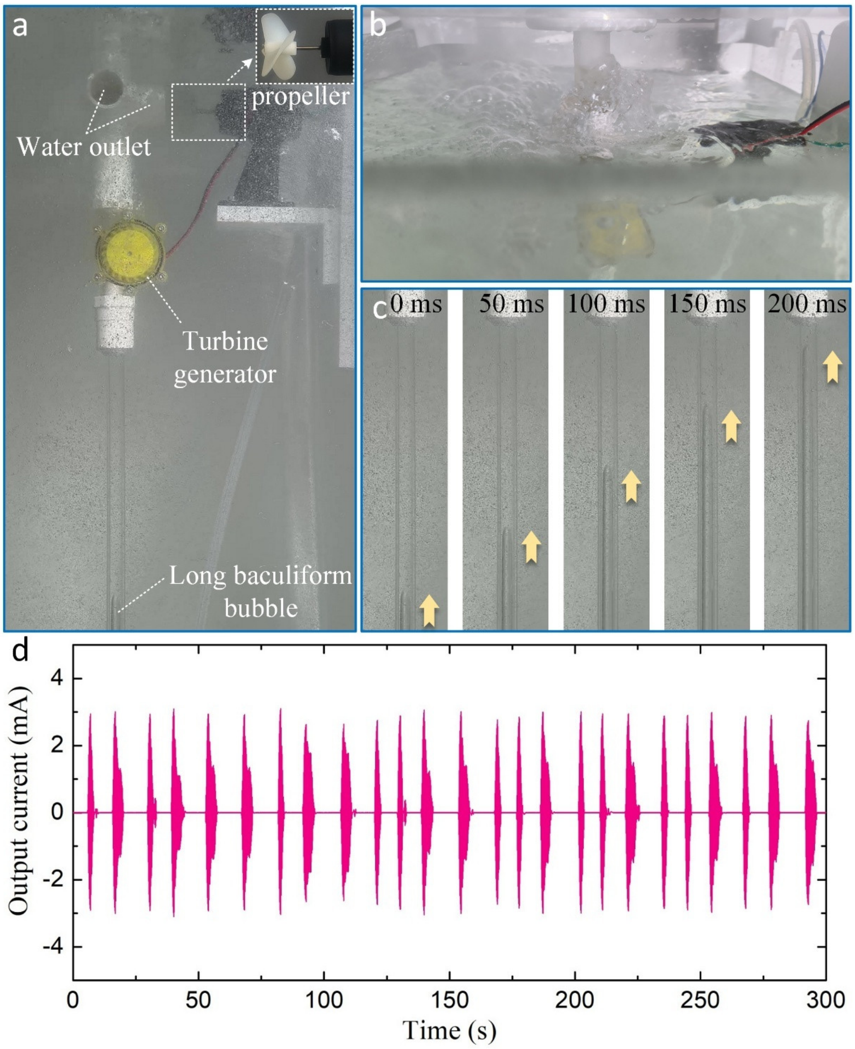

Figure S11 Stability test of the bubble energy harvesting device under external turbulent disturbances. (a) Turbulent disturbance generated by the rotation of an underwater propeller. (b) Vigorous undulating motion of the water surface. (c) Fluid motion state in the bubble rising pipe of the energy harvesting device under external turbulent disturbances. (d) Output current of the energy harvesting device at a gas flux of 70.1 mL·min^{-1}.

Despite the intense turbulent disturbances in the external water environment, the bubble

energy harvesting device still maintains a stable working state, which is reflected in the motion state of the fluid in the pipe and the electrical output of the device. In terms of the fluid motion state, as shown in Figure S11(c), during the bubble-release stage, the flow pattern in the pipe remains a stable slug flow, where a long baculiform bubble sufficient to fill the pipe diameter rises rapidly in the pipe. It proves that the bubble rising pipe of the device and the external water environment still maintain the stable water circulation depicted in Figure S3. In terms of electrical output, the peak output current of the device in each bubble-release stage stabilizes at approximately 3 mA, and the number of current peaks within 300 s is 25, which is similar to the electrical output results without external turbulent disturbances shown in Figure 5d of the main text. By virtue of the shielding effect of the bubble rising pipe, the flow direction of the fluid inside the pipe always aligns with the pipe axis and is not disturbed by the complex fluid motion in the external environment, thus ensuring that the water flowing through the turbine generator always moves along the pipe axis (tangential to the turbine). The fluid inside the pipe of the energy harvesting device always maintains a stable state of directional flow and electrical output performance. These experimental results confirm that the bubble energy harvesting system based on the mechanical self-adaptive porous valve exhibits favorable anti-disturbance ability and practical application potential.

12.2 Bubble plume impingement

To demonstrate the stability of the aerophobic characteristics of the passive porous interface mechanical structure, an underwater bubble plume aeration impingement test was conducted on the porous valve. The experimental setup is shown in Figure S12(a), where a bubble plume releaser with a diameter of 9.6 cm is placed below the gas reservoir of the porous valve. The bubble plume generator is connected to an air pump via a silicone hose to release microbubbles with diameters on the micrometer scale into the water. The bubble plume releaser outputs highly dense microbubbles with a high specific surface area at a gas flux of 22.5 $mL \cdot min^{-1}$ to the gas reservoir of the porous valve. After the bubble plume impingement test was completed, the contact angle of a bubble on the surface of the porous plate in the porous valve was measured and presented in Figure S12(b). The open-circuit voltage of the energy harvesting device during the continuous impingement process of the bubble plume was measured and plotted in Figure S12(c).

As shown in Figure S12(b), after 10000 s of continuous impingement by highly dense bubble plumes, the bubble contact angle on the surface of the passive porous interface mechanical structure in seawater is 149°, which is close to the pre-impingement contact angle of 146° (Figure S2), demonstrating the stable aerophobic characteristics of the porous structure. As depicted in Figure S12(c), the open-circuit voltage of the bubble energy harvesting device based on the porous valve consistently exhibits stable periodic variations, corresponding to the cycle of bubble accumulation and high-speed release in the porous valve. During the 10000-second experiment, the porous valve released bubbles approximately every 17 seconds. The peak values of the open-circuit voltage over nearly 600 bubble-release cycles remain stable at around 0.2 V. Notably, the impingement intensity of bubble plumes in the actual ocean is far lower than the test conditions in this experiment. The gas flux of bubbles released per unit area of the seafloor is extremely low in real marine environments. For example, the gas fluxes at two observed bubble seepage points in

the cold-spring activity area of Site F in the South China sea are 6.75 and 2.18 mL·min^{-1}, with corresponding bubble diameters of 3.25 and 2.54 mm, respectively. This indicates that the gas flux, specific surface area, and impingement intensity of bubble plumes in actual marine environments are all much lower than the conditions of this experiment. Despite such harsh test conditions, the aerophobic characteristics of the passive porous interface mechanical structure and the electrical output of the energy harvesting device remain stable. These results validate that the proposed porous valve maintains a stable operational state, thereby ensuring that the bubble energy harvesting system based on the porous valve exhibits stable output performance.

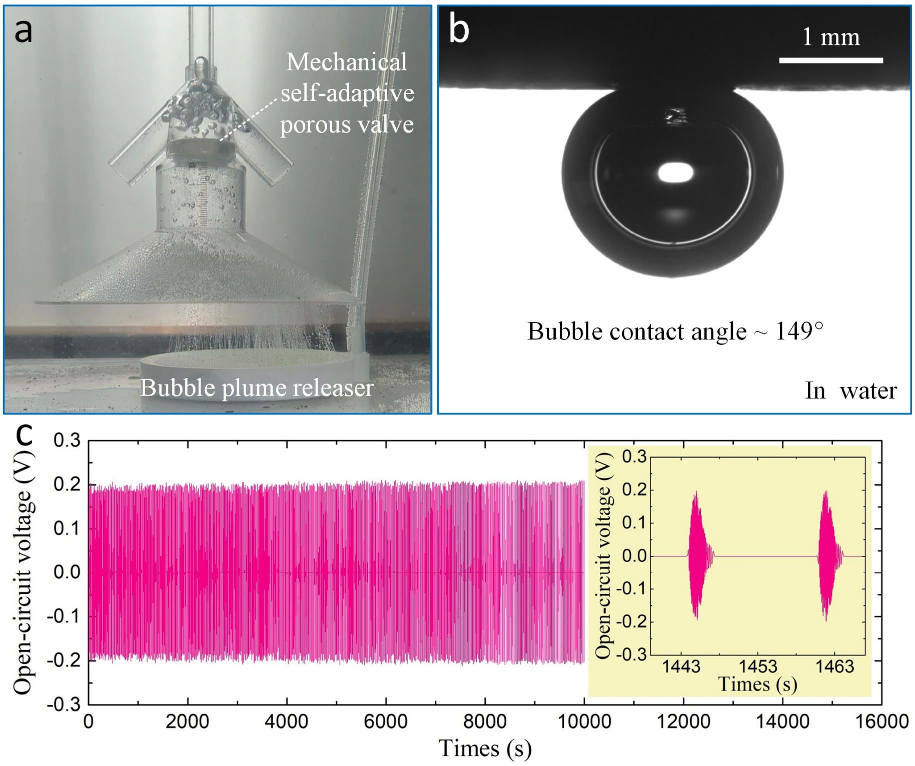

Figure S12 Stability test of the bubble energy harvesting device based on the porous valve under bubble plume aeration impingement. (a) Aeration experimental setup; (b) Bubble contact angle on the surface of the passive porous interface mechanical structure in the porous valve after continuous scouring by bubble plumes; (c) Open-circuit voltage of the bubble energy harvesting device over 10000 s. The inset is the current waveforms in 1439–1467 s.
