# Supplementary material for: Mechanical self-adaptive porous valve relying on surface tension for energy harvesting from low-flux bubbles
Source: Nat Commun. 2025 Nov 22;16:11544. doi: 10.1038/s41467-025-66500-6 (PMC12749621; doi:10.1038/s41467-025-66500-6)
Supplement: Supplementary file 2 — Description of Additional Supplementary Files [file 41467_2025_66500_MOESM2_ESM.pdf]

Description of Additional Supplementary Files

Supplementary Movie 1

Description: This Movie records the physical process in which underwater bubbles continuously accumulate in the gas reservoir of the mechanical self-adaptive porous valve and are then released at high speed through numerous micropores. In the movie, the pore diameter of the porous plate in the porous valve is 0.8 mm. The playback speed of the Movie is 0.25 times the normal speed. This movie demonstrates the morphological changes of the bubbles during the process of passing through the numerous micropores.

Supplementary Movie 2

Description: This Movie shows the phenomenon that the gas-liquid interfaces in all conical micropores gradually protrude in an upward direction during the process of bubble accumulation beneath the porous valve with 1.5-mm pores. This phenomenon confirms the interface mechanical principle shown in Figure 1d in the main text. During the process from bubble accumulation to release, the radius of curvature of the gas-liquid interface in each micropore first decreases and then increases. Consequently, under the action of surface tension, the Laplace pressure of the gasliquid interface undergoes an adaptive change that first increases and then decreases. Such self-adaptive mechanical characteristics provide the mechanical conditions for the accumulation and high-speed release of bubbles.

Supplementary Movie 3

Description: This Movie records the process of bubble accumulation and release in the porous valves with different pore diameters to study the threshold characteristics of the porous valves. The experimental phenomena show that the opening threshold and the maximum accumulated gas volume of the porous valves decrease as the pore diameter increases, which confirms the results of the theoretical analysis of interfacial mechanics.

Supplementary Movie 4

Description: This Movie shows the performance of the bubble energy harvesting device without a porous valve at different gas fluxes. When the gas flux is 11.0, 32.3, and 41.9 mL·min-1, the turbine generator above the bubble rising pipe cannot rotate. This experimental phenomenon proves that the bubble energy harvesting device without the porous valve cannot work under low-gas-flux conditions, while the device equipped with the porous valve is not limited by gas fluxes.

Supplementary Movie 5

Description: This Movie demonstrates the energy harvesting and utilization of the ultralow-flux photosynthetic bubbles released by Riccia fluitans by the energy harvesting device based on the porous valve. The photosynthetic bubbles accumulated in the porous valve enter the bubble rising pipe rapidly after reaching the threshold. During the process of bubble rising, the buoyancy potential energy of the bubbles is converted into the energy of the gas-liquid two-phase fluid in the pipe and then converted into electrical energy by the turbine generator and stored into a capacitor. The collected bubble energy is successfully used to drive an underwater temperature sensor. The underwater bubble energy harvesting device based on the porous valve can provide in situ energy for sensors in underwater environment monitoring.
